# Extraction and characterization of keratin from bovine hoof: A potential material for biomedical applications

**DOI:** 10.1186/2193-1801-3-596

**Published:** 2014-10-10

**Authors:** Prachi Kakkar, Balaraman Madhan, Ganesh Shanmugam

**Affiliations:** Central Leather Research Institute, Council of Scientific and Industrial Research, Adyar, Chennai, 600020 India

**Keywords:** Proteins, Keratin, Biocompatibility, α-helix, β-sheet, Biopolymers & renewable polymers

## Abstract

Keratin from the hoof is a less explored source for making valuable products. In this paper we present the extraction of pure keratin from bovine hooves and characterized them to better address the possible exploitation of this bio-resource as an alternative material for tissue engineering applications. The keratin protein from the pulverized hooves was extracted by reduction, which was observed to be pure, and two polypeptide chains of molecular weight in the range of 45–50 and 55–60 KDa were determined using SDS-PAGE assay. FTIR analysis complementing circular dichroism (CD) data, established that hoof keratin predominantly adopted α-helical conformation with admixture of β-sheet. The keratin was shown to have appreciably high denaturation temperature (215°C) as indicated by differential scanning calorimetric (DSC) analysis. Thermogravimetric analysis (TGA) also showed the retention of 50% of the original weight of the sample even at a temperature of 346°C. The keratin from the hoof had been observed to be biocompatible when analyzed with MTT assay using fibroblast cells, showing more than 90% cell viability. Hence, hoof keratin would be useful for high value biomedical applications.

## Introduction

A broad category of insoluble proteins that associate as intermediate filaments (IFs), a cytoskeletal element with 8–10 nm diameter, were being referred to the term “keratin”. Apart from being the principal constituent of the stratum corneum of human and animal skin, keratins are also found in exoskeletal materials such as horns, hooves, hair, feathers, wool and nails (Aluigi et al. 2007; Gupta and Nayak 2014). Keratins (mammalian) were categorized into two distinct groups namely hard and soft, based on their structure, function and regulation. Hard keratins form ordered arrays of IFs embedded in a matrix of cystine rich proteins and contribute to the tough structure of epidermal appendages, whereas soft keratins preferentially form loosely-packed bundles of cytoplasmic IFs (Coulombe et al. 2000; Fraser et al. 1986; Moll et al. 1982) and typically contain less sulphur (Zoccola et al. 2009). There are also variations in the secondary structure of the keratin from these sources. As observed earlier (Hill et al. 2010; Iridag and Kazanci 2006; Zoccola et al. 2009), mammalian keratins are found to be predominantly of the α-type (contains α-helical structure), whereas birds and reptiles can possess both α- and β-types (contain a mixture of α-helical and β-sheet structures). Fraser and Parry (Fraser and Parry 1996) suggested that the inner regions of compact assemblies of keratin molecules are predominantly hydrophobic, and that charged residues are being concentrated at the surface. Characteristically, keratins show high stability and low solubility due to –S–S– cross-linking between cysteine amino acid residues (Brandelli 2008; Feughelman 2002). The most distinctive feature of keratin at a molecular level is the high concentration of half-cystine residues (7%–20% of the total amino acid residues), most of which are localized at the terminal regions of the proteins (Hearle 2000).

Huge amount of keratin byproducts are wasted without ample utility (Korniłłowicz-Kowalska and Bohacz 2011). Several attempts have been made for the mechanical (Korol 2012) as well as microbial utilization of keratin wastes (Chaudhari et al. 2013; Fang et al. 2013; Jeong et al. 2010). Keratin like other natural biopolymers viz., collagen and chitosan can be used for making biomaterials in tissue repair and regeneration (Alsarra 2009; Natarajan et al. 2012; Ramshaw et al. 2009; Ramadass et al. 2013). Such applications would enhance the value of utilization of keratins, as recent reports support the acceptance of keratin as a material for biotechnology and biomedical applications (Hill et al. 2010; Rouse and Van Dyke 2010; Zhuang et al. 2013; Patrucco et al. 2011).

Cell adhesion sequences, RGD (Arg-Gly-Asp), and LDV (Leu-Asp-Val), which are found in the extra cellular matrix proteins such as fibronectin, are present in the keratins of wool, silk, and human hair (Feughelmann 1985; Marshall et al. 1991). Moreover, keratin also contains cellular-binding motifs (i.e. super secondary structure with binding capacity) which mimic the sites of cellular attachment found in the native extra cellular matrix because of which, keratin could be used for the development of tissue engineering constructs. Recently, Srinivasan et al. (Balaji et al. 2012) reported the use of keratin from horn for biomaterial development. Although keratin has been extracted from many sources such as wool, hair, feather, horn etc., the source of bovine hoof, which is a solid waste from the slaughter houses, is less explored. Hence, this paper deals with the extraction of high value keratin from bovine hoof. The extracted keratin from hoof has been characterized to establish its structure and biocompatibility.

## Materials and methods

Pulverized raw hooves were obtained from the local slaughter house at Perambur, Chennai (India). This material was washed thrice with distilled water to remove all dirt and then drained. After drying it completely in an oven, it was used as raw material for further studies. All other chemicals used for analytical purpose were purchased from Sigma-Aldrich.

### Extraction of keratin

The first step of extraction is defatting i.e. removal of fats from the raw material. Soxhlet’s apparatus was used to carry out defatting/delipidization of pulverized hoof sample for about two days. Mixture of hexane and dichloromethane (1:1, v/v) was used for refluxing. Ten gram of defatted hoof sample was mixed with 7 M urea, 6 g SDS and 15 ml of 2-mercaptoethanol in a 300 mL round-bottom flask and kept in orbital shaker at 60°C for 12 h to extract keratin at pH 7. The resulting solution was then centrifuged for 15 mins at 6,000 rpm and the supernatant was dialyzed against degassed water for 5–6 days. Some of the extracted keratin was kept in a deep freezer at -80°C for 5 h and lyophilized to make it into powder. Hereafter, the dialyzed keratin i.e. prior to lyophilization will be mentioned as liquid keratin and the powdered one will be mentioned as lyophilized keratin.

### Quantification of protein

The protein-content was determined through CHNS analysis. Carbon, hydrogen, nitrogen and sulfur contents of the lyophilized keratin sample were analyzed using varioMICRO CHNSO Sr no: 15091002. Based on this analysis along with the available literature of amino acids present in hoof keratin, protein content in the sample was quantified.

To determine the molecular weight, the liquid keratin was denatured prior to subjecting it to one-dimensional slab SDS-PAGE (10% gel) by heating the sample mixed with an equal amount of sample buffer containing SDS and β-mercaptoethanol. In the beginning, it was at kept at 50 V and then switched to 100 V once the protein (sample) just reached the separating gel. The concentration of liquid keratin used was about ~30 μg per well. The gel was stained using coomassie brilliant blue stain (Neuhoff et al. 1985).

### CD Spectroscopy

CD spectrum of keratin was recorded at 25°C on a JASCO J-715 spectropolarimeter using 1 mm rectangular quartz cell. The sample was prepared by dissolving lyophilized keratin in 50 mM phosphate buffer (pH 5). The sample concentration was 0.1 mg/ml. The CD spectrum (θ) was recorded from 190 to 260 nm with standard sensitivity and at the scan rate of 100 nm/min. The other parameters such as band width, response, and data pitch were set at 1 nm, 1 sec, and 0.5 nm, respectively. The CD spectrum represents an average of three individual scans. After subtracting the CD spectrum of solvent (50 mM phosphate buffer, pH 5) from the sample spectrum, it was converted to specific ellipticity [Ѱ]_λ_ using the following formula:
1

Where, θ is an observed ellipticity, C is the concentration of sample in g/ml and ℓ is the cell path length in cm.

### FTIR Spectroscopy

FTIR spectra of keratin were recorded from 400 to 4000 cm^-1^ using a Nicolet 20 DXB FT-IR spectrophotometer. For solid-state measurement, a pellet was prepared by mixing lyophilized keratin with potassium bromide. For film measurement, an aqueous buffer solution (pH 5) containing keratin (5 mg/ml) was cast on ZnSe plate. The sample was kept at room temperature until a dry thin film formed on the surface of ZnSe. All spectra were measured at a resolution of 8 cm^-1^.

### Thermal analysis

DSC analysis of lyophilized keratin after conditioning (for 24 hrs) the samples at 24°C, 65% relative humidity was performed from 30°C to 250°C, at 10°C/min using universal V4.4A TA instruments. The instrument was calibrated by an indium standard, and the calorimeter cell was flushed with 100 mL/min liquid nitrogen.

TGA of lyophilized keratin was performed on universal V4.4A TA instruments. The sample was heated at 10°C/min at a temperature range of 30°C-600°C using Al_2_O_3_ crucibles.

### MTT Assay for cell viability

3T3-Li fibroblast cells were used for biocompatibility analysis. The cells were cultured in flask containing Dulbecco’s Modified Eagle’s Medium (DMEM; Sigma) with 10% Fetal Bovine Serum (FBS; Invitrogen) supplemented with 50 U/ml penicillin, 50 μg/ml streptomycin and 2.5 μg/ml amphoterecin B. To determine the biocompatibility of the extracted keratin, the cells were cultured as monolayer in polystyrene cell culture 96-well plate with and without keratin at 37°C under a humidified atmosphere of 5% CO_2_ in air. Different concentrations of the keratin used for the biocompatibility assay were 10, 25 and 50 μg/well.

## Results and discussion

### Extraction of keratin

**Figure 1:**
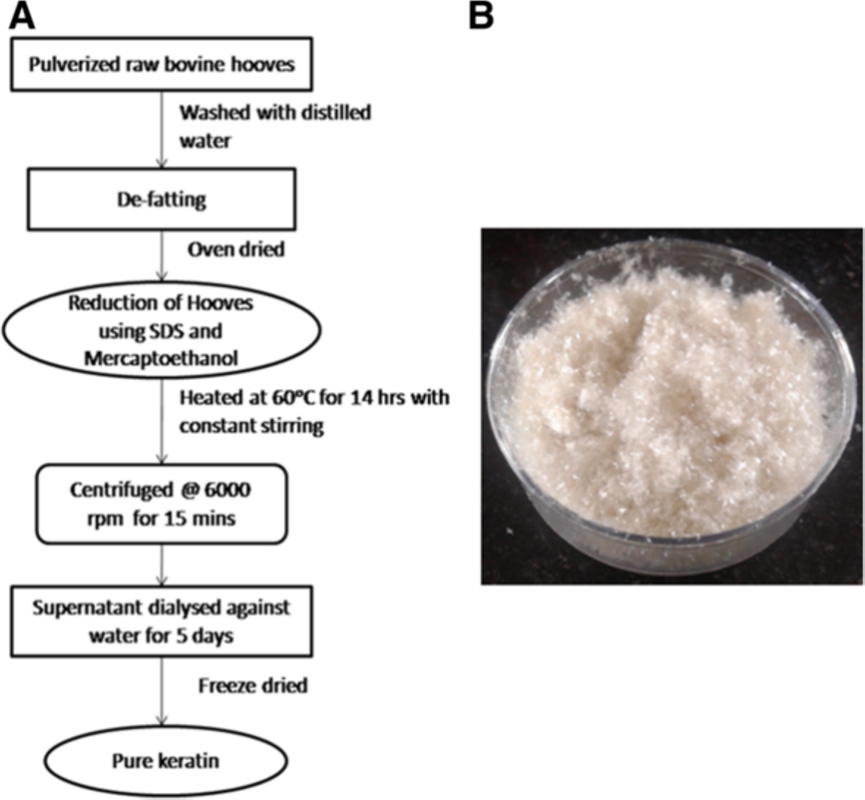
**Extraction of Hoof Keratin. (A)** Flow chart describing the extraction procedure. **(B)** Lyophilized pure keratin.

While a significant amount of research carried out on several sources of keratin, there are scanty reports available on the extraction, characterization and utilization of keratin from bovine hooves. Keratin from hooves is a significant source along with other sources of keratin. Here we report the extraction and characterization of keratin from the much un-explored raw material- bovine hoof in the reduced form by adopting a well established method by Yamauchi et al. (Yamauchi et al. 1996). Keratin was obtained in the form of an aqueous solution from raw hooves using urea (to break non-covalent bonds), sodium dodecyl sulfate (for disruption of strong intermolecular interactions), and mercaptoethanol (to cleave the disulfide bonds in keratin), at 60°C. The process followed for the extraction of keratin is presented in the flow chart given in Figure 1A. The optimum pH range adopted for extraction was 6–8, as keratin could not be extracted at acidic pH and is likely to undergo decomposition at alkaline pH. This solution is expected to be stable for 1 year when stored at ambient temperature (20°C-24°C) (Srinivasan et al. 2010). The lyophilized powder of this keratin solution is shown in Figure 1B.

### Quantitative estimation of keratin

The quantitative estimation of keratin was initially determined by both Lowry’s and Bradford’s method of protein estimation. But, the results shown by both these analyses were not accurate and constant. This might be due to the presence of some traces of mercaptoethanol in the final dialysate. Hence, nitrogen content was determined using CHNS analyzer. The lyophilized keratin sample contained 13.3% of nitrogen, 45.3% of carbon, and 6.84% of hydrogen. Based on the amino acid analysis of the horn-hoof reported earlier (Zoccola et al. 2009), the nitrogen content of the keratin is 16.7%. Higher amount of hydrogen in our sample does not rule out the possibility of the presence of bound water attributing towards the constituent of the sample. Hence the purity of the keratin sample is observed to be greater than 80%. Based on the initial weight of the hooves, 44% yield of keratin was obtained.

**Figure 2:**
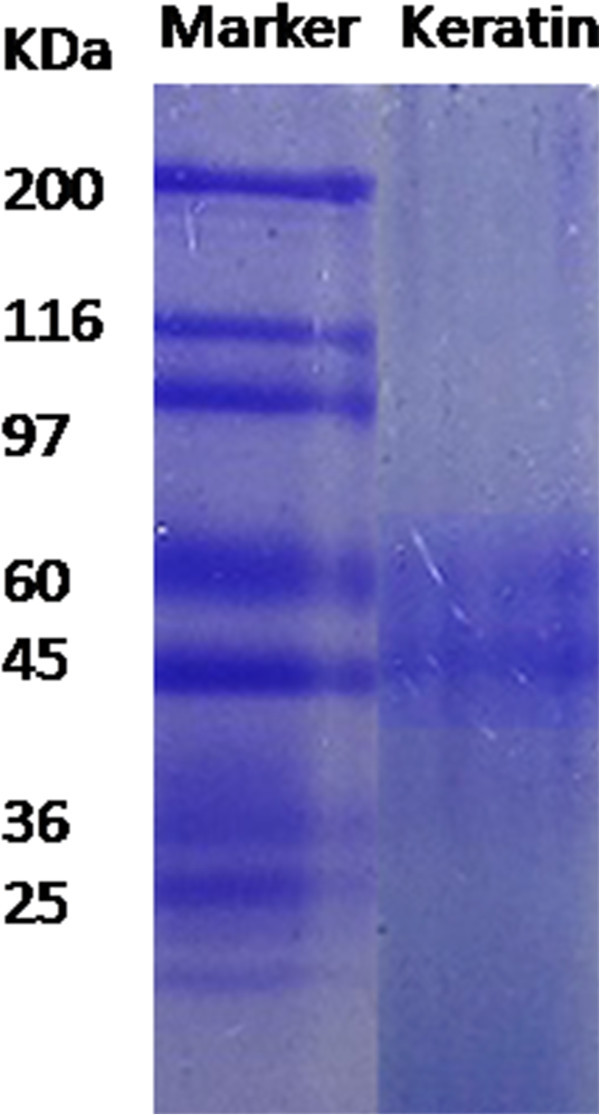
**SDS-PAGE of standard protein molecular weight markers (left lane) and hoof keratin (right lane).**

Molecular weight of the keratin had been estimated by SDS-PAGE using 10% polyacrylamide gel, which showed two clear protein fractions with equal intensities between 45–50 and 55–60 kDa as shown in Figure 2. These bands are similar to the low-sulphur keratin observed earlier (Fraser and Parry 1996).

### CD analysis of keratin

**Figure 3:**
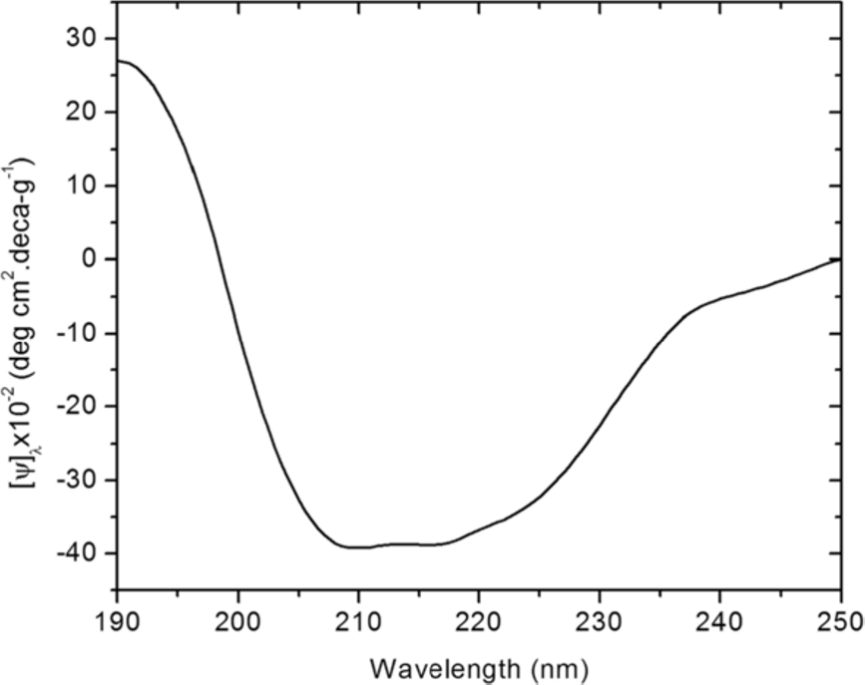
**CD spectrum of hoof keratin.**

CD is a quick method to elucidate the secondary structure of protein and peptide in solution. By observing the wavelength and sign of the emerging bands, the kind of secondary structure (α-helix, β-sheet, or random coil etc.) can be evaluated. Generally, an α-helical structure shows two negative minimums of almost same degree of ellipticity at 222 nm and 208 nm followed by a positive maximum at 190 nm while a negative minimum near 218 nm followed by a positive maximum at 195 nm depicts the presence of β-sheet structure (Kelly et al. 2005). In the present case (as shown in Figure 3), the CD spectrum shows the negative bands at 208 nm and shoulder at 222 nm, indicating that hoof keratin adopts predominantly α-helical conformation. However, the intensities of these two negative bands are not equal and also CD spectrum has broad band between 200 and 235 nm, suggesting that keratin also adopts β-sheet conformation (Amiya et al. 1982). Hence, hoof keratin is comprised of predominantly α-helical conformation with an admixture of β-sheet in liquid state.

### FTIR analysis of keratin

The secondary structure of keratin was also analyzed using FTIR spectroscopy. Similar to CD, FTIR provides the information about the secondary structure of protein in solution. However, FITR can be directly used in solid and film states compared to CD where special modification requires in the instrument to avoid artifacts (Kuroda et al. 2001). Consequently, the secondary structure of hoof keratin was examined in both film, derived from aqueous solution, and solid states. Similar to CD, FTIR exhibits characteristic bands for α-helix, β-sheet, β-turn, and random coil conformations in the amide I (1700–1600 cm^-1^) and amide II (1560–1500 cm^-1^) regions (Kong and Yu 2007). Among these regions, amide I (due to the C = O stretch vibrations of the peptide linkages) is more sensitive to protein secondary structures. It is well established that α-helical conformation has an amide I and II (mainly from in-plane N-H bending and from the C-N stretching vibration) bands between 1657 and 1650 cm^-1^ and between 1550 and 1540 cm^-1^, respectively (Lyman et al. 2001; Wojciechowska et al. 2000), while the β-sheet has an amide I and II bands between 1635 and 1615 cm^-1^ and between 1535 and 1520 cm^-1^, respectively (Goormaghtigh et al. 2006).

**Figure 4:**
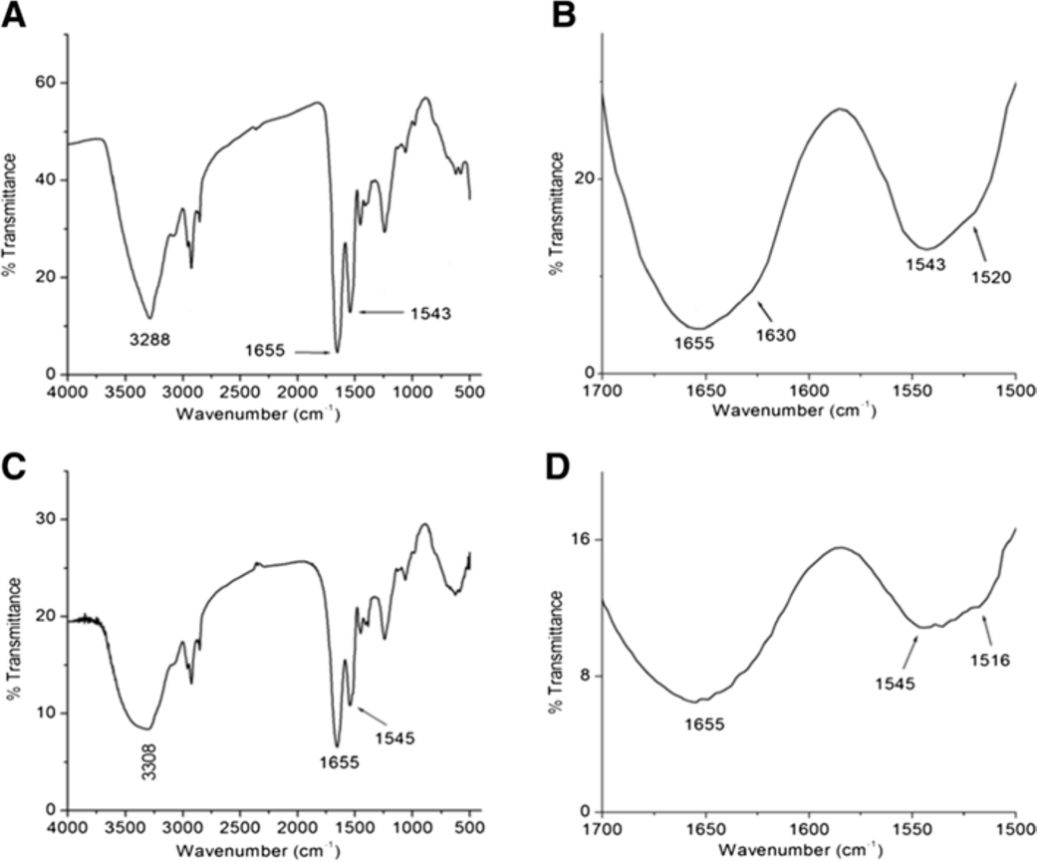
**FTIR spectra of hoof keratin in the film (A) and solid (C) states. (B)** and **(D)** represent the expanded region (amide I and II) of the corresponding FTIR spectra.

The FTIR spectrum of keratin in solid (lyophilized) and supported film (liquid) states are shown in Figure 4. The keratin film, derived from aqueous buffer solution (pH 5), shows an amide I and II bands at 1655 and 1543 cm^-1^ (Figure 4A), respectively, which are characteristic of α-helical conformation. However, the expanded region clearly shows an additional shoulder band at 1620 cm^-1^ in the amide I region and shoulder band at 1520 cm^-1^ in the amide II regions, which are the signature of β-sheet conformation (Figure 4C). The FTIR result thus clearly suggests that keratin adopts predominantly α-helical conformation with admixture of β-sheet conformation in the supported film state. Similar to supported film, the appearance of amide I and II bands at 1655 and 1545 cm^-1^ (Figure 4B), respectively, indicates that keratin adopts predominantly α-helical conformation in the solid state. Again, the broadness of amide I band and the appearance of weak amide II bands at 1516 cm^-1^ (Figure 4D) suggest that keratin also adopts admixture of β-sheet conformation in the solid state. These results reveal that the secondary structure of keratin in the film and solid states are similar. Further, the FTIR result, along with solution CD, demonstrates that the secondary structure of hoof keratin in solid and film states are similar to that of solution state.

Unlike amide I and II, the N-H stretching vibration (3310–3270 cm^-1^) of peptide bond (-CO-NH-) is insensitive to the conformation of the polypeptide backbone as it is exclusively localized on the NH group. But the frequency of N-H stretching depends on the strength of the hydrogen bond. In the present case, the characteristic frequency of hydrogen bonded N-H has been observed at 3288, and 3308 cm^-1^ for keratin in both film and solid states, respectively, confirming the folded conformation of keratin. Hence both CD and FTIR confirm that the extracted keratin contains ordered structure and is not in denatured form. This keratin having an admixture of α helix and β sheets, seems to have structural similarity with the keratins in birds and reptiles.

### Thermal analysis

**Figure 5:**
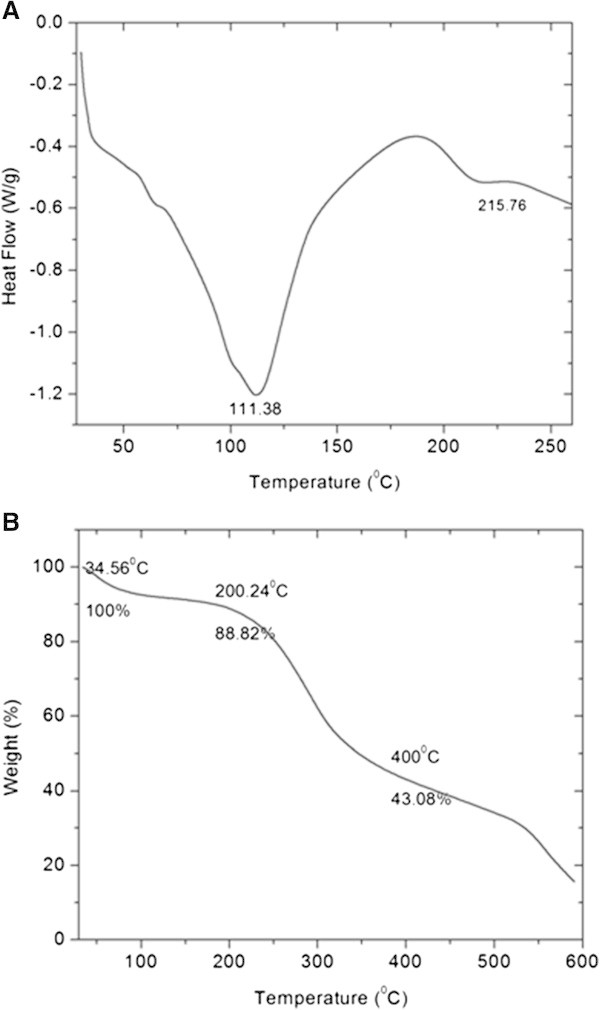
**Thermal analysis of hoof keratin (A) DSC profile (B) TGA profile.**

DSC is the most popular thermal technique, which measures the heat absorbed or liberated during various transitions in the sample due to temperature treatment. The DSC thermogram of keratin is shown in Figure 5A.

The denaturation temperature observed around 111°C is due to water evaporation [38]. The endothermic peak observed above 200°C is reported for helix denaturation in keratin [39]. The second endothermic peak for the hoof keratin is observed around 215°C. Appreciably high thermal stability of the biomaterial would be an advantage for applications where thermal stability is important. Hence, keratin has an obvious advantage in relation to its thermal stability compared to some of the commonly used biopolymers viz., collagen.Thermogravimetric analysis was carried out to quantify the thermal degradation of keratin in a controlled atmosphere. The TGA curve for keratin sample is shown in Figure 5B. The initial weight loss of around 12% observed between 100°C and 150°C is due to the loss of bound water associated with keratin. Presence of 12% moisture substantiates that the purity of keratin sample could be greater than 90% based on the composition of nitrogen and water. The weight loss between 200 and 400°C is due to the breakdown of polypeptides and associated degradation. More than 50% weight loss was observed at 400°C.

### Biocompatibity assay

**Figure 6:**
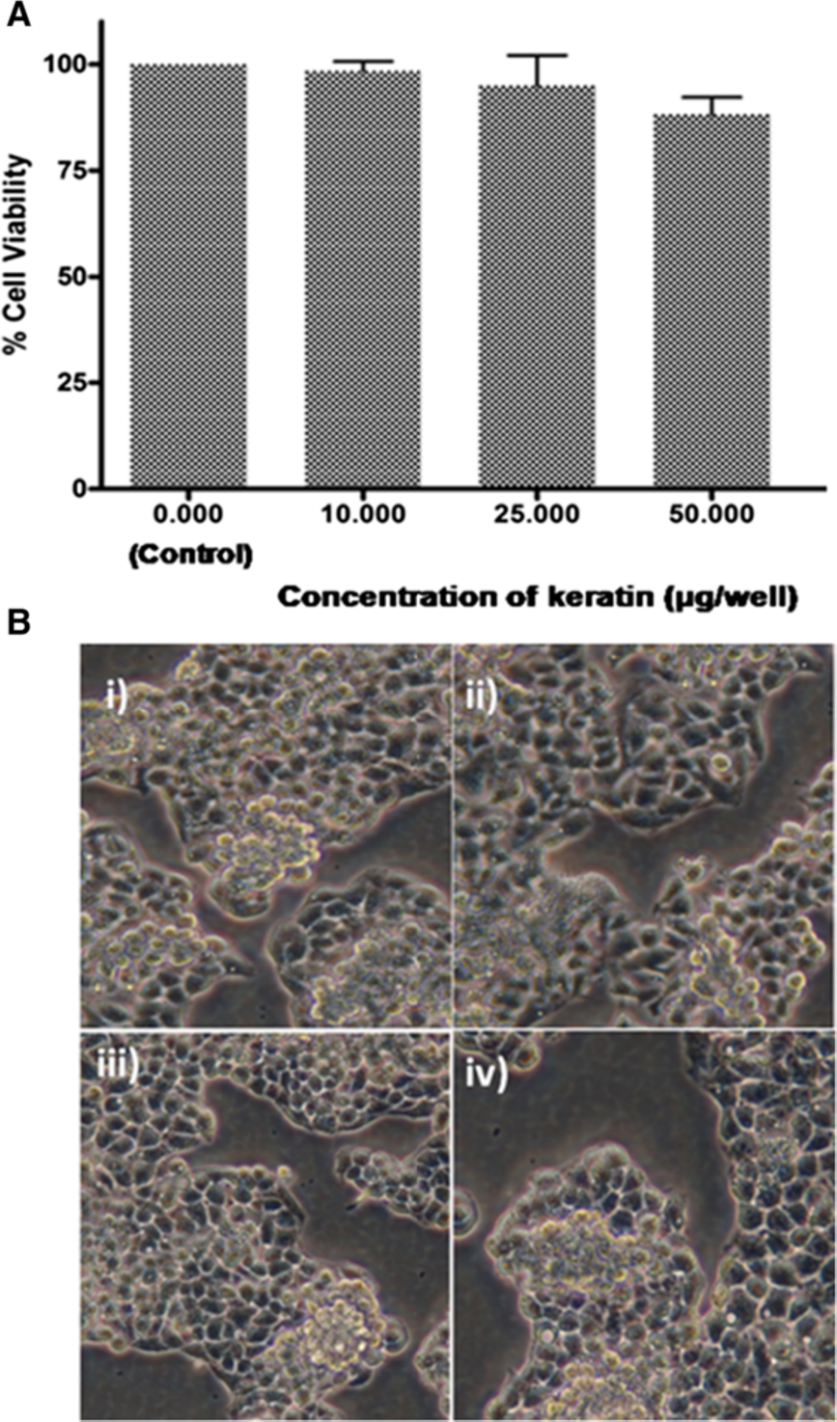
**Biocompatibility of the extracted hoof keratin (A) MTT assay using 3 T3 fibroblast cells and (B) the growth of fibroblasts cell shown in the optical micrographs for (i) Control cells- 3 T3-fibroblastic untreated cells with normal aggregation (ii) 10 μg/well treated cells showing syncytium formation (iii) 25 μg/well treated cells with distinct cell membranes eiv) 50 μg/well treated cells.**

The measurement of cell viability and growth is essential to establish the biocompatibility of biomaterials. The MTT assay, a colorimetric assay is employed for measuring the cell viability, involving the reduction of MTT reagent, giving a purple color formazan product in the presence of viable cells. Thus, the absorbance of formazan product proportionally reflects the measure of viable cells. To analyze the biocompatibility of hoof keratin, MTT assay was carried out at different concentrations of keratin (μg/well). It showed good cell viability with reference to control (untreated) as depicted by Figure 6.The figure shows the images of immature fibroblast cells. We can observe normal aggregation and formation of actin filaments in control (untreated), where cells were allowed to grow on polystyrene plate. When the concentration of keratin was increased from 10 to 25 μg/well, cell membranes are observed to be disctinct and internal boundaries are segregated. Even at concentration of 50 μg/well of keratin, normal cell attachement can be observed. From Figure 6, it is clear that hoof keratin is a biocompatible material and hence it can find wide-spread applications as one of the alternative materials for tissue engineering applications.

## Conclusions

In this work we show that pure keratin biopolymer can be extracted from bovine hooves. Characterization of the hoof keratin suggests that, structurally, it belongs to the category of birds and reptiles keratins as it contains a mixture of α- helical and β-sheet structure. Hoof keratin possesses high thermal stability. MTT assay using fibroblast cells shows that the hoof keratin is a biocompatible material for promoting cellular attachment. Hoof keratin appears to be new and promising source of biomaterial for biomedical and tissue engineering applications.
